# Supplementary material for: Divergent functions of late ESCRT components in Giardia lamblia: Insights from subcellular distributions and protein interactions
Source: PLoS Negl Trop Dis. 2025 Nov 12;19(11):e0013700. doi: 10.1371/journal.pntd.0013700 (PMC12611102; doi:10.1371/journal.pntd.0013700)
Supplement: S3 Table — (DOCX) [file pntd.0013700.s003.docx]

**S3 Table: List of Gene ID and UniProt ID used in this study**

| Gene name | Gene ID | Uniprot ID |
| --- | --- | --- |
| *glist1* | Gl50803_0011129 | A8BUY9 |
| *ScIST1* | YNL265C | P53843 |
| *ScVPS4* | YPR173C | P52917 |
| *glvps4b* | Gl50803_16795 | A8BUC0 |
| *HsVps4B* | 9525 | O75351 |
