# Supplementary figures and images for: Divergent functions of late ESCRT components in Giardia lamblia: Insights from subcellular distributions and protein interactions
Source: PLoS Negl Trop Dis. 2025 Nov 12;19(11):e0013700. doi: 10.1371/journal.pntd.0013700 (PMC12611102; doi:10.1371/journal.pntd.0013700)

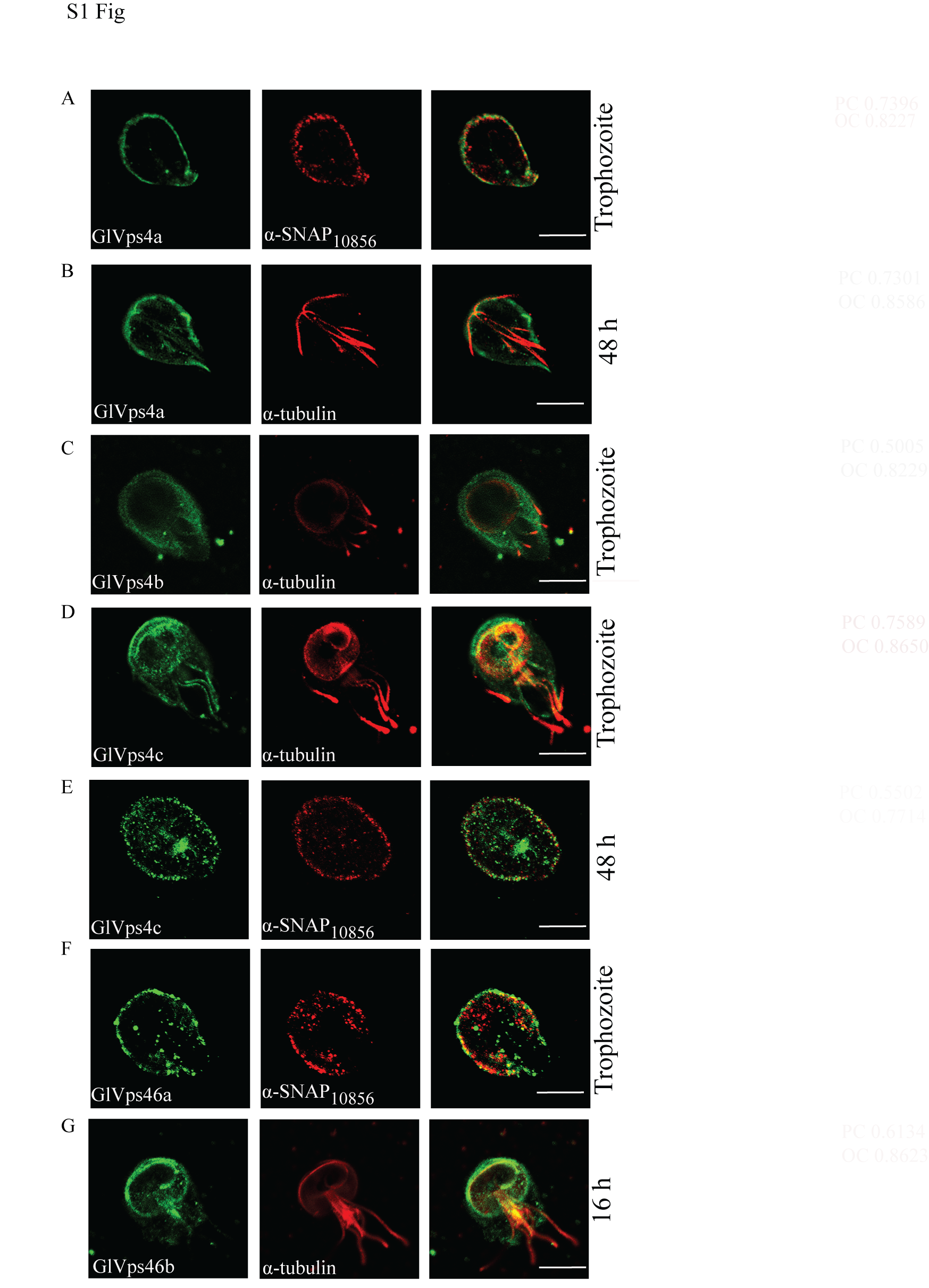

Supplement: S1 Fig — (A) GlVps4a (green) with α-SNAP10856 (red) in trophozoites. (B) GlVps4a (green) with α-tubulin (red) at 48 h post-encystation. (C) GlVps4b (green) with α-tubulin (red) in trophozoites. (D) GlVps4c (green) and α-tubulin (red) in the trophozoites. (E) GlVps4c (green) with α-SNAP10856 (red) at 48 h post-encystation. (F) GlVps46a (green) with α-SNAP10856 (red) in trophozoites. (G) GlVps46b (green) with α-tubulin (red) at 16 h post-encystation. Merged images show colocalization in yellow. Scale bar: 8 µm. (TIF) [file pntd.0013700.s005.tif]

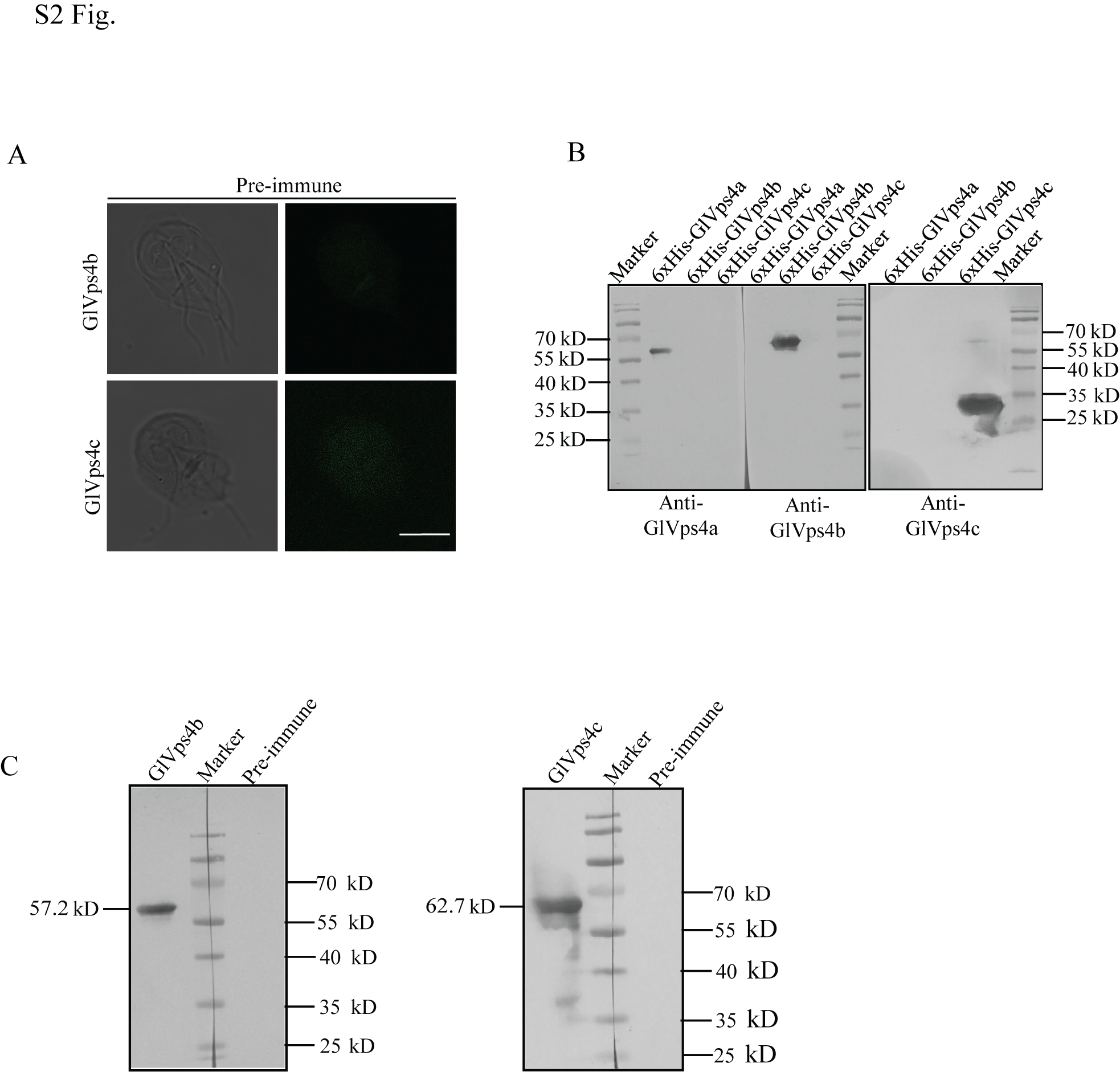

Supplement: S2 Fig — (A) Cells treated with pre-immune sera collected from animals prior to immunization with GlVps4b (upper panel) or with GlVps4c (bottom panel). Scale bar 8 µm. (B) Western blotting of the three 6x-His tagged GlVps4 paralogues expressed in E. coli and then purified from the bacterial extracts. Blots were incubated with anti-GlVps4a antibody (left), with anti-GlVps4b antibody (middle), and with anti-GlVps4c antibody (right). The detections of bands of sizes ~ 56kDa, ~ 60 kDa, and ~30k Da demonstrate the specificity of GlVps4a, GlVps4b, and GlVps4c antibodies, respectively. (C) Western blots were performed using trophozoite extracts. The blot was developed using the anti-GlVps4b antibody (left), while the blot was developed using anti-GlVps4c (right). (TIF) [file pntd.0013700.s006.tif]

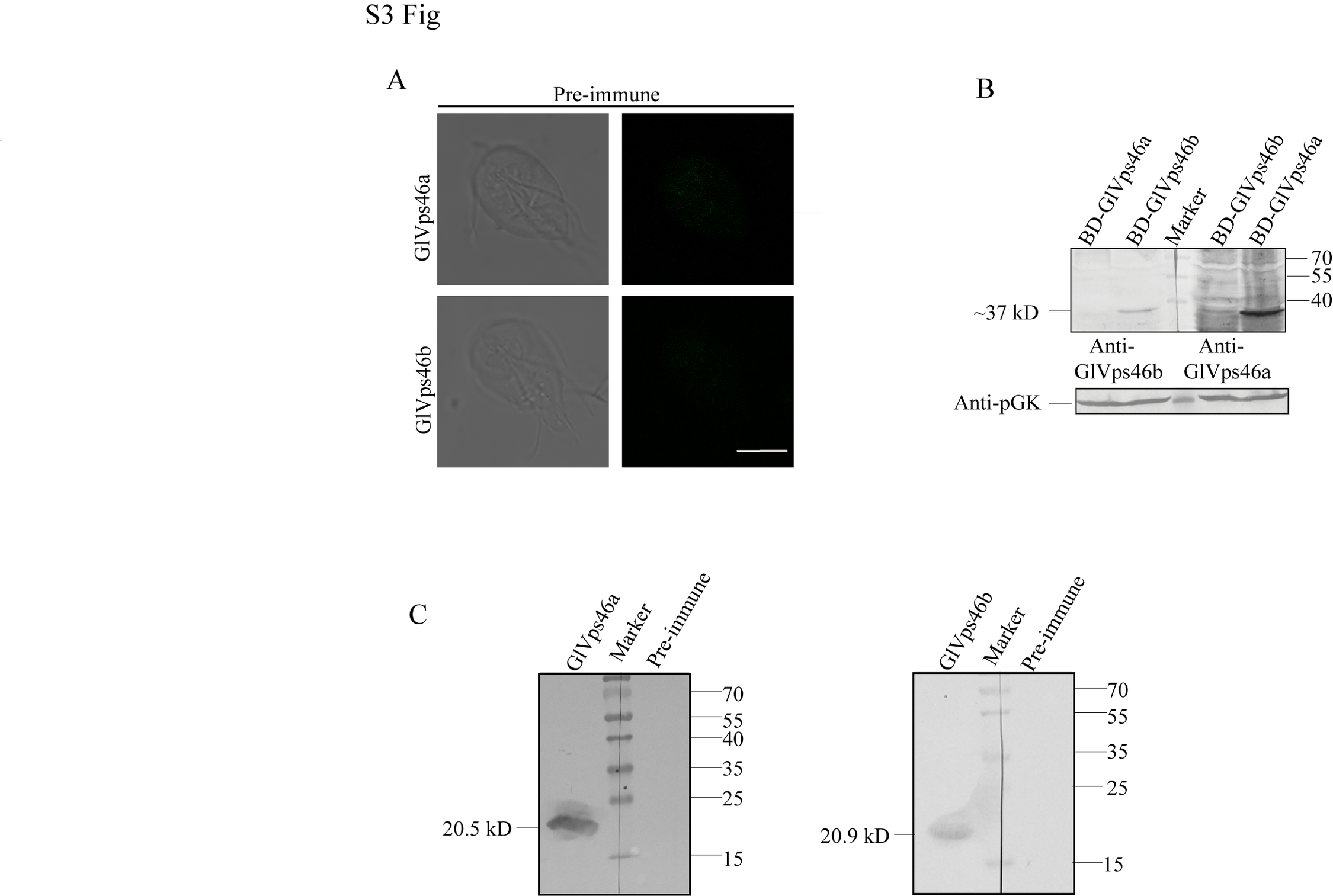

Supplement: S3 Fig — (A) Cells treated with pre-immune sera were collected from animals prior to immunization with GlVps46a (upper panel). Cells treated with pre-immune sera were collected from animals prior to immunization with GlVps46b (bottom panel). Scale bar 8 µm. (B) Western blot analysis was performed using PJ69-4A transformants expressing either BD-tagged GlVps46a or BD-tagged GlVps46b. The western blot was performed with anti-GlVps46b antibody (left) and anti-GlVps46a antibody (right). A ~ 37.4 kDa band (right) and a ~ 37.8 kDa band (left) were detected. (C) Western blots were performed using trophozoite extracts. The blot was developed using the anti-GlVps46a antibody (left) and anti-GlVps46b antibody (right). All western blots were performed using prestained protein ladder from Thermo Scientific (26616) markers except for GlVps46b (26619). (TIF) [file pntd.0013700.s007.tif]

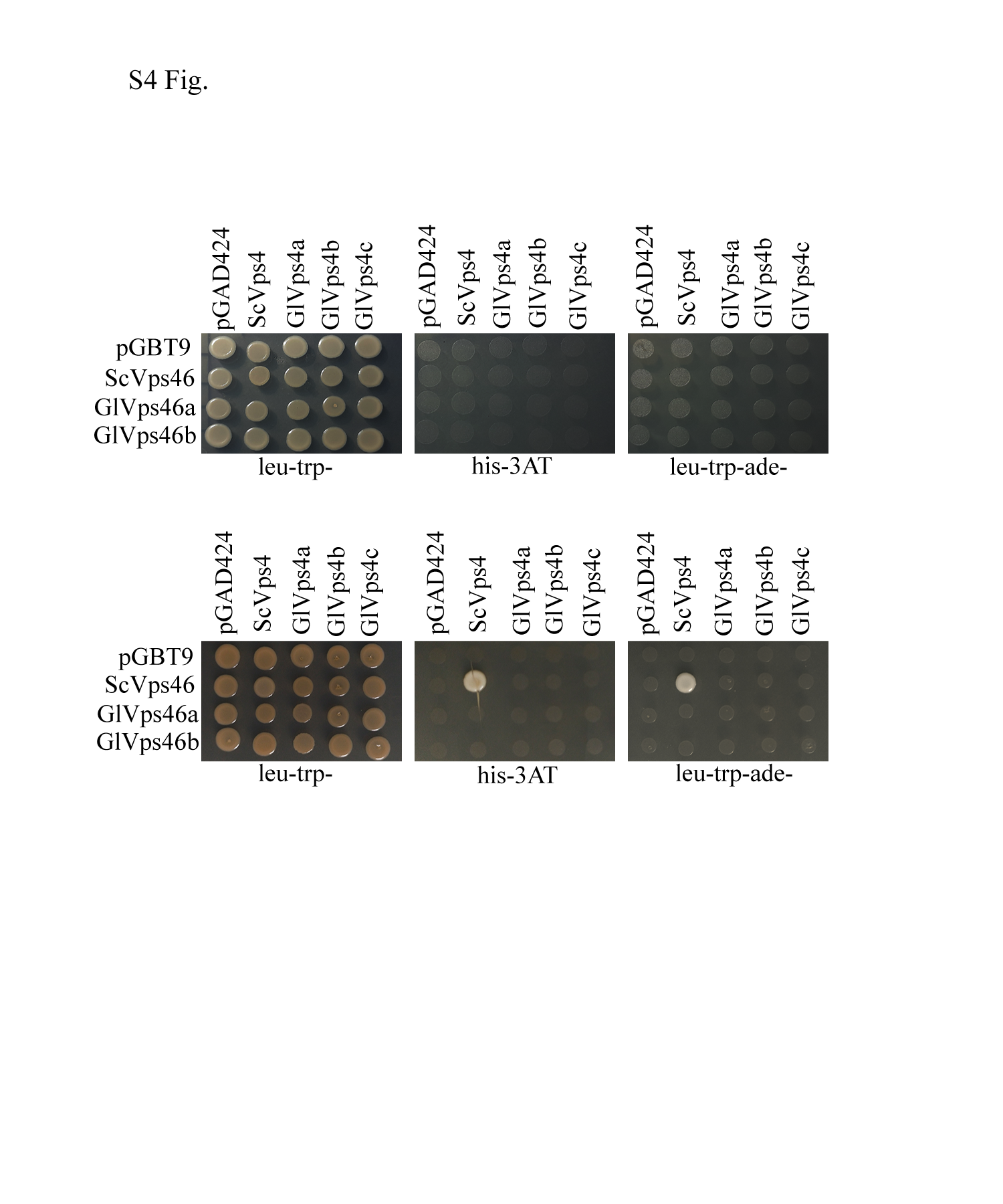

Supplement: S4 Fig — PJ69-4A cells were transformed with various BD and AD fusion combinations, as indicated in the figure. Growth of the PJ694-A transformants were monitored on SD leu-trp- (left panel), SD leu-trp-his- with 2.5 mM 3-AT (middle panel), and leu-trp-ade- (right panel). The experiment was repeated with constructs expressing proteins with reversal of BD or AD fusion (bottom panels). (TIF) [file pntd.0013700.s008.tif]

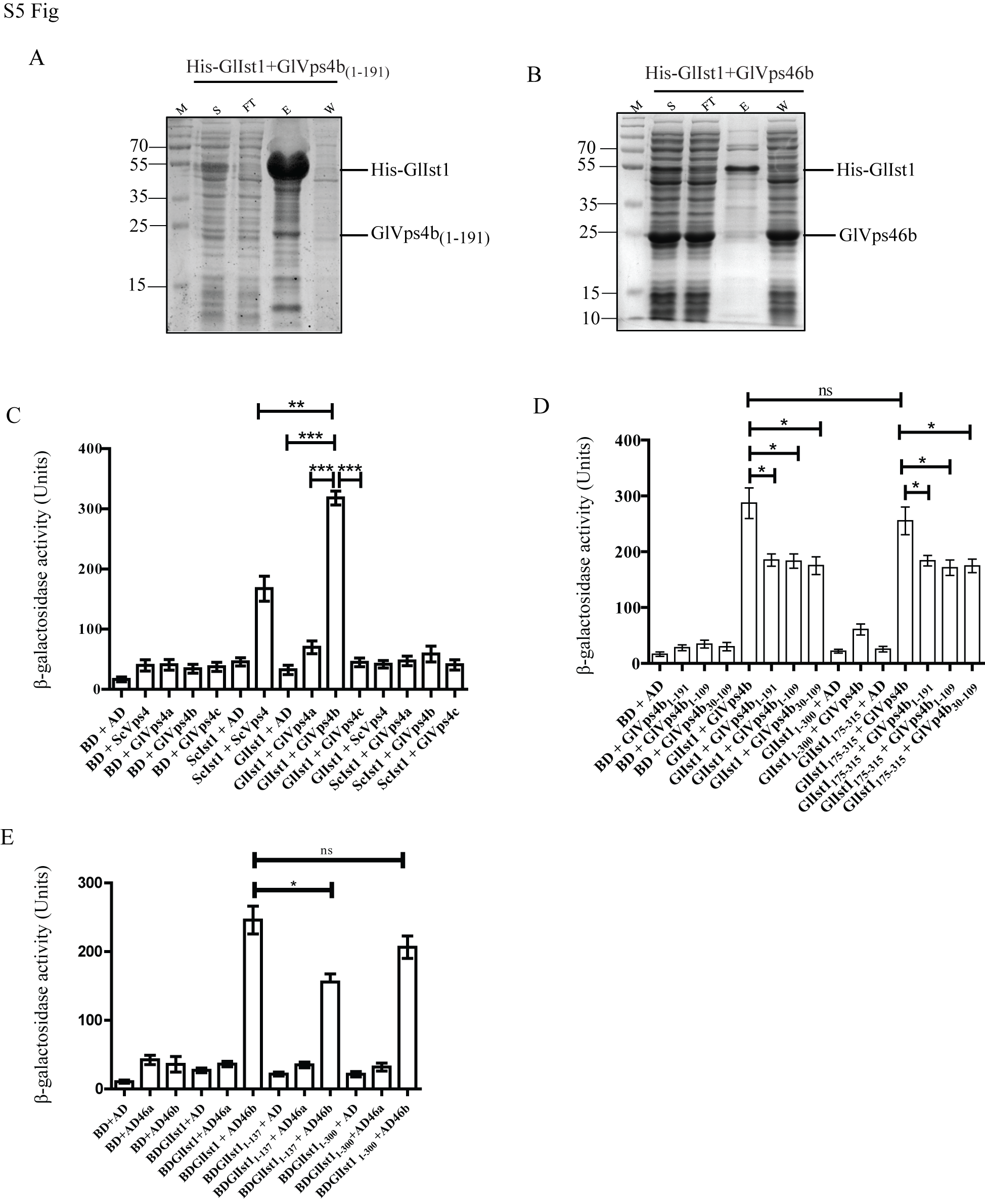

Supplement: S5 Fig — (A) 12% SDS-PAGE showing co-elution of GlIst1 along with GlVps4b1–191 in the elution fraction. (B) 12% SDS-PAGE showing co-elution of GlIst1 along with GlVps46b in the elution fraction, S: total supernatant; FT: flowthrough; E: elution; W: wash. (C) An extended representation of quantification of β-galactosidase activity shown in Fig4A, (D) Fig 4E and (E) Fig 5B. (TIF) [file pntd.0013700.s009.tif]

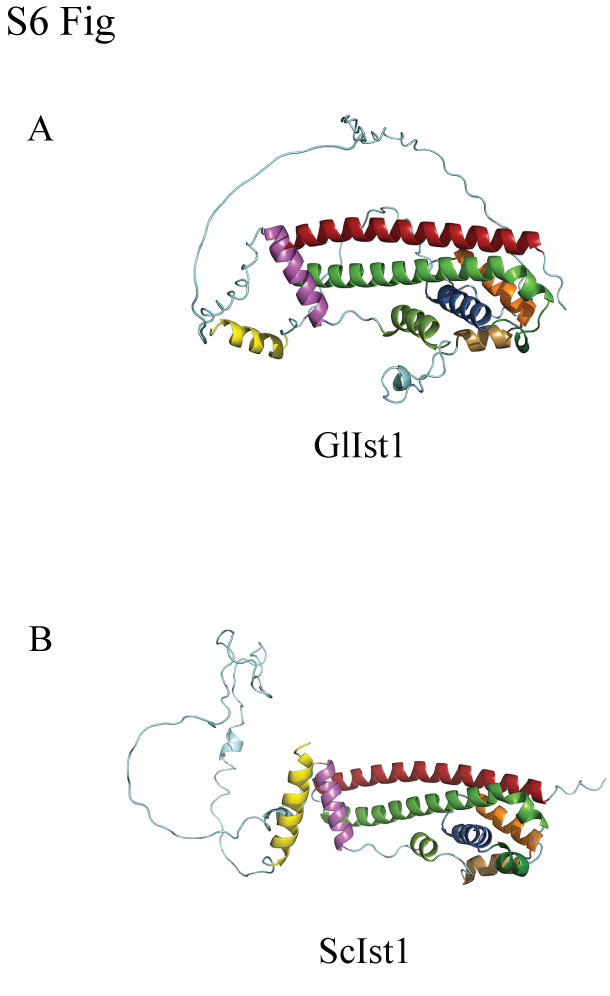

Supplement: S6 Fig — (A) GlIst1 of Giardia lamblia. (B) Ist1 of S. cerevisiae. The helices are colour-coded to indicate their position relative to the N-terminus: α-1 (red), α-2 (green), α-3 (blue), α-4 (orange), α-4a (sand), α-4b (chartreuse green) and α-5 (magenta) and the MIM (yellow). The unstructured regions are in cyan. (TIF) [file pntd.0013700.s010.tif]

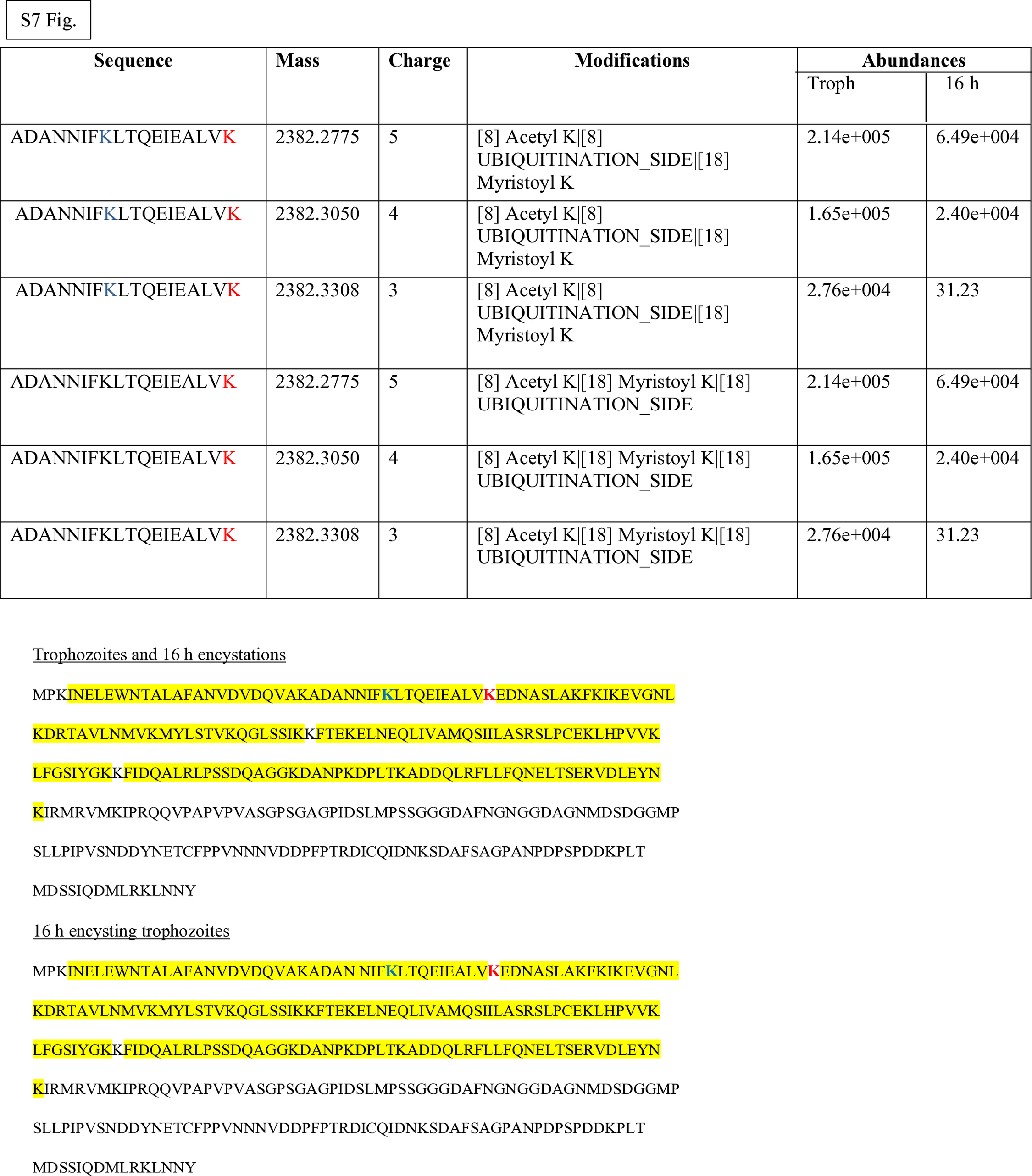

Supplement: S7 Fig — (A) Peptide fragments of GlIst1 showing myristoylation (red) and ubiquitination (blue). (B) Overall detection of peptide fragments is highlighted in yellow, with the myristoylated K residue highlighted in red. The peptide sequence coverage for GlIst1 was 55.8%. (TIF) [file pntd.0013700.s011.tif]

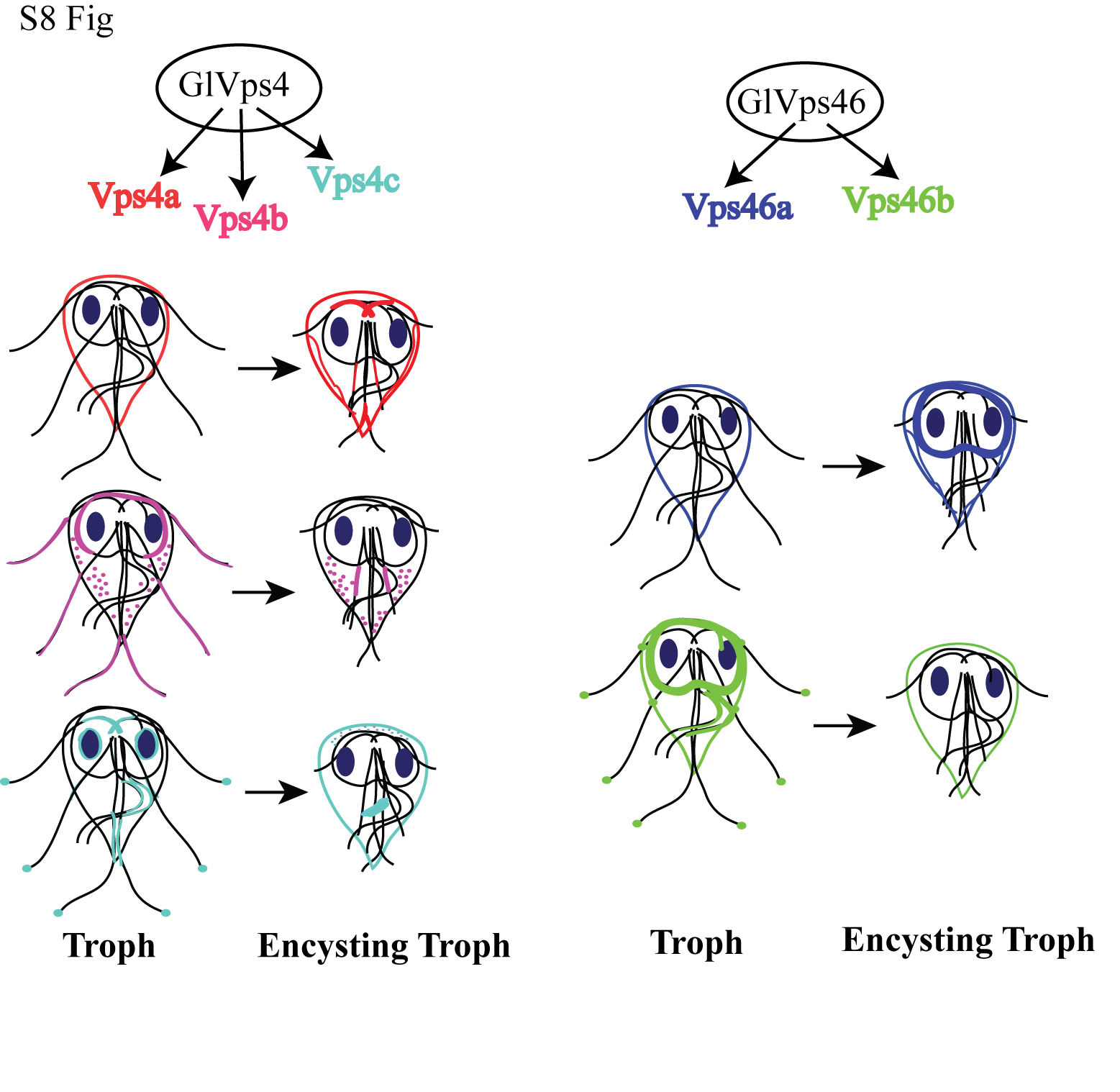

Supplement: S8 Fig — (TIF) [file pntd.0013700.s012.tif]
