# Supplementary material for: Divergent functions of late ESCRT components in Giardia lamblia: Insights from subcellular distributions and protein interactions
Source: PLoS Negl Trop Dis. 2025 Nov 12;19(11):e0013700. doi: 10.1371/journal.pntd.0013700 (PMC12611102; doi:10.1371/journal.pntd.0013700)
Supplement: Attachment — Submitted filename: Response to reviewers.rtf [file pntd.0013700.s014.rtf]

Reviewer 1
Are the figures (Tables, Images) of sufficient quality for clarity? I believe that the quality of the figures and legends can be improved to make them easier for the reader to understand. For example, it would be positive to increase the cellular structures where each of the molecules was found. I also suggest a scheme that explains in cartoon form the participation of each of the ESCRT paralogs studied here.
Authors response:
We appreciate the reviewer's valuable feedback regarding the figure clarity. Kindly note that upon submission, the system-generated PDF version of the manuscript may have reduce the resolution of embedded figures. To ensure optimal clarity and allow detailed examination, we are happy to provide the original high-resolution TIFF files of all the figures upon request. As per the Reviewer's suggestion, we have included a cartoon illustrating the distribution of each Vps4 and Vps46 paralog in trophozoites and during encystation (S8 Fig).  We thank the reviewer for this helpful suggestion. 

Are the limitations of the analysis clearly described? No
Authors response:
In the Discussion section, we outline prospective experimental approaches aimed at elucidating the functional divergences between the paralogous proteins (Line 679).  

Although the two-hybrid experiments are convincing, I consider that double-labeling experiments to observe and study the interaction of Vps4 and Vps46 paralogs would provide more evidence about their different locations in the cell, which would allow us to hypothesize about their function. Furthermore, western blot assays would reinforce the identity of the proteins
Authors response:
We thank the reviewer for this thoughtful suggestion. We agree that double-labeling experiments would provide important additional insights into the interaction and sub-cellular localization of Vps4 and Vps46 paralogs. But this line of investigation was not possible as all the antibodies used in this study were raised in rabbit. The reason for this limitation stemmed from the fact that the study commenced just prior to COVID pandemic, thereby limiting access to the host institute's vivarium.  The situation forced us to take the services of a contract research organization who could only use rabbits. Unfortunately, due to resource constraints we are unable to raise new antibodies and perform these additional experiments within the scope of the present revision. However, we have noted this valuable suggestion and plan to incorporate these approaches in our future studies to further investigate the localization and functional interactions of these proteins. Regarding the reviewer's second point, western blot analyses of GlVps4 and GlVps46 paralogs have already been performed to confirm the expression and identity of the proteins. These data are included in the Supplementary S2 and S3 Fig.

Reviewer 2

Are the limitations of the analysis clearly described? No
Authors response:
In the Discussion section, we outline prospective experimental approaches aimed at elucidating the functional divergences between the paralogous proteins (Line 678).  

A rationale should be provided as to why encysting trophozoites are observed specifically at 16h and 48h post-induction
Authors response:
We have incorporated justification for the 16 h (Line 297) and 48 h (Line 301) timepoints, which we hope the reviewer will consider to be adequate.

The authors analyze the subcellular distribution of GlVps4 paralogs in trophozoites and after encystation induction. At 16 h, GlVps4a was detected in the PVs, axonemes of the anterior flagella (AF), and other locations. How have the authors determined such specialized intracellular structures such as PV, AF, VD and C inside the parasite without any counterstaining with such structure-specific markers? 
Authors response:
To substantiate the claim that GlVps4a, GlVps4c and GlVps46a localize to the PVs, we performed colocalization with á-SNAP_10856_ (S1 Fig).  We have previously reported that this á-SNAP localizes to the PVs in trophozoites, where it remains even during encystation [1]. 
Next, to substantiate our claim that GlVps4a, GlVps4b, GlVps4c and GlVps46b are associated with various flagella and the ventral disc, all of which are constituted by microtubules, we performed colocalization with anti-alpha-tubulin. (S1 Fig).
We do not have access to a suitable cytosolic marker at present to perform co-localization. Our conclusion is support by a previous study showing a similar distribution of a cytoplasmic protein, carbamate kinase (Gl50803_16453) [2].

Line 330: The authors claim that "all paralogs of GlVps4 localize to microtubule-rich structures." However, microtubules were not stained. To validate this statement, a co-localization study should be performed.
Authors response:
As mentioned above, we have performed co-localization with á-tubulin as a marker for microtubules. These experiments, as described in the revised manuscript (S1 Fig), demonstrate that all three paralogs of GlVps4, showed co-localization with á-tubulin, indicating their association with microtubule-rich structures at different stages of its life cycle. 

Line 346: If the data supporting this statement is not shown, then it should be removed.
Authors response:
The line has been removed.

Lines 367-368: To substantiate the claim of increased GlVps46a signal, qPCR should be performed to analyze its expression pattern. 
Authors response:
As suggested, we have performed quantitative PCR (qPCR) to analyse the expression pattern of glvps46a under different encysting conditions. The qPCR result suggests, at both 16 h and 48 h of encysting trophozoites, the expression of glvps46a was significantly increased compared to the non-encysting (0 h) trophozoites. The increase in expression was statistically significant. However, it is important to note that changes in subcellular localization of proteins can occur independently of transcriptional regulation. Similar phenomena have been previously observed in Giardia for other proteins such as GlRpn10, where significant subcellular redistribution took place without marked transcriptional changes [3]. Thus, we think qPCR will not be effective in explaining the increased signal intensity at VD in encysting trophozoites compared to trophozoites, observed by IF. Additionally, we have not included the qPCR data in main manuscript as going forward we may use this in an independent study.


 


To confirm the interaction of GIIST1 with GIVps4b or GIVps4b6b, a co-immunoprecipitation (Co-IP) in a homologous system
Authors response:
While we fully appreciate the importance of confirming protein-protein interactions in a homologous system, we could not perform co-immunoprecipitation (Co-IP) in the homologous system due to the lack of a specific antibody against GlIst1. To overcome this technical limitation, we have demonstrated the interaction between GlIst1 and both GlVps4b and GlVps46b using co-purification assay (Fig 4D and 5B). 

Line 564-571: the relevant figure or supplementary figure number should be cited. If Figure 6B is being mentioned, how did the authors confirm that GlIS1 localizes to both the endosome and vacuole membranes? The authors should carry out immunostaining against an endosome/vacuole-specific marker to substantiate their claims.
Authors response:
We apologize for this oversight.  The relevant figure has been cited in the revised manuscript (Line 579-586).  This section has been restructured as per reviewers' suggestions, including colocalization in live yeast cells with endosome and vacuole membrane markers.

The authors examined the GlIst1-GlVps4b interaction in a heterologous system. Yeast two-hybrid is known to be sticky in nature and thereby generate lot of protein-protein interaction artifacts. It would be better if these claimed interactions are validated in homologous system.
Authors response:
Please see previous explanation stating constraints regarding lack of anti-GlIst1 antibody limiting our ability to perform co-immunoprecipitation (Co-IP) in the homologous system as suggested, and how we have used co-purification experiments to validate the interactions of GlVps4b_1-191_ or GlVps46b with GlIst1. The GlVps4b_1-191_ fragment was successfully co-eluted with His-tagged GlIst1, confirming their interaction. Thus, our results support the interaction of the N-terminal domain of GlVps4b with GlIst1 by both yeast two-hybrid and copurification (Fig 4). 

The authors, using LC-ESI-MS/MS analysis, observe that the K43 residue undergoes a unique myristoylation. It is not described how out of different possibilities they arrive at myristoylation. Additionally, it would be helpful if they also use other biochemical methods, such as radiolabeled myristate or a click chemistry-based technique etc to substantiate their claim.
Authors response:
Three biological replicates were used to analyse the data.  Only peptides that were detected at least three times in all three independent runs and exhibited high signal intensity were retained for downstream analysis, ensuring robustness in peptide identifications.  To minimize ambiguity in protein identification, only the mapped peptides were used for quantification and calculation of protein sequence coverage of GlIst1. Sequence coverage was calculated as the percentage of its full-length amino acid sequence that was represented by these identified and uniquely mapped peptides.  
While our data did reveal other post-translational modifications such as ubiquitination, we chose to highlight myristoylation due to its potential biological significance in the context of ESCRT protein membrane recruitment. Existing literature records that certain ESCRT proteins associate with membrane via lipidation.  In the revised manuscript, we have mentioned that membrane recruitment can be facilitated by lipid modifications such as myristoylation, palmitoylation and prenylation. Though palmitoylation and prenylation was not detected, the residue K43 was found to be myristoylated. Additionally, we have mentioned about ubiquitination at K33 in our revised manuscript (Line 591-594).
We acknowledge the reviewer's suggestion to further validate myristoylation using complementary biochemical approaches such as radiolabeled myristate incorporation or click chemistry-based assays. At present, our laboratory facilities are not equipped to conduct radioactive labeling experiments or click chemistry validations. However, we consider this a valuable direction for future work and will pursue such biochemical validations as we expand our experimental capabilities.

Contradictory Statements in Lines 349-351: The claim that there are "MORE cytoplasmic puncta" yet the corresponding figure shows that the "cytoplasmic localization is diminished". This appears to be contradictory hence clarification is needed. 
Authors response:
We apologize for this confusion, which was due to oversight. We have now updated the text to accurately convey our findings (Line 355-357). 

Minor Comments
1. Scale bars are absent in Figures 1A, 1B, 2A, 2B, and 6B.
2. The unit "hour (h)" is missing after "48" in line 288. Please correct this.
3. 'trophozoite' panel should be added to Figure 1A for comprehensive visualization.
4. Line 300: The statement, "ESCRT machinery is known to induce membrane curvature…" lacks a citation. A relevant reference should be added to support this claim.
5. Lines 346-348: The source of this statement should be explicitly stated or referenced.
6. The Y-axis labels in Figures 4A and 4D is missing.
7. In lines 552-553, the relevant figure or supplementary figure number should be cited for the LC-ESI-MS/MS data.
Authors response:
All minor comments have been addressed.


1. 	Datta SP, Jana K, Mondal A, Ganguly S, Sarkar S. Multiple paralogues of á-SNAP in Giardia lamblia exhibit independent subcellular localization and redistribution during encystation and stress. Parasites & Vectors. 2018 Oct 4;11(1):539. 
2. 	Balmer EA, Wirdnam CD, Faso C. A core UPS molecular complement implicates unique endocytic compartments at the parasite–host interface in Giardia lamblia. Virulence. 2023 Dec 31;14(1):2174288. 
3. 	Sinha A, Datta SP, Ray A, Sarkar S. A reduced VWA domain-containing proteasomal ubiquitin receptor of Giardia lamblia localizes to the flagellar pore regions in microtubule-dependent manner. Parasites Vectors. 2015 Dec;8(1):120. 


Reviewer 3

Major comments:
On the first half of the paper, authors successfully identified the localization of GIVps4 and 46 paralog proteins (Fig. 1&2). These findings give new knowledge in the field. On the other hand, the function of these proteins was not assessed in their experiments. Nevertheless, authors emphasized functional importance of these proteins over the paper, which seems very speculative, not logical interpretation of their results. In the latter half, authors tried to address the interaction between GlIst1 and GlVps4/46 paralogs (Fig. 3-5). They successfully identified that only specific paralogs (GIVps4b and GIVps46b) can interact with GlIst1, in which they also identified the binding sites of the activity. These data beautifully identified the interaction between them. Finally, they found post-translational modification, myristoylation of Ist1 (Fig. 6). However, their impact on Giardia lifecycles was not presented, as well.
Overall, authors identified some important findings about GIVps4 (or 46) in Giardia, however, further experiments, such as blockade, knockdown, or overexpression, are required for their conclusions and discussions. The reviewer felt authors should add some data for assessing functions of these proteins, or restructure overall descriptions in the manuscript as less speculative style before considering the formal assessment for acceptance.
Authors response:
We thank the reviewer for the suggestion regarding the direct functional validation of the identified late-ESCRT components. We agree that targeted knockdown or overexpression experiments would greatly strengthen the functional conclusions. However, at present, time and resource limitations are significant impediments for such experiments.  Instead, as per the reviewer's suggestion, we have restructured the Results section to eliminate all speculative statements and limit their use even in the Discussion section.  We believe these changes better align the manuscript's inferences with the data presented while still showcasing the important new insight about the ESCRT machinery in G. lamblia.
As mentioned in the Discussion, we are in the process of performing knockdown, and other functional assays to dissect the precise roles of these late-ESCRT components during Giardia stage-conversion and lifecycle progression. These longer-term studies will add more insights into the ESCRT-mediated cellular processes.

Other comments:
1. In figure 1&2 and related descriptions, localization of GIVps4/46 were explained by the positional relationship to the organelles, such as PV, FL, PF, CF, and AF, however, these organelles were presented by the morphology on DIC, not by the immunofluorescence staining. Also, image resolution seems not enough to tell the exact localization of GIVps4/46 in the organelle.
Authors response:
In response to the reviewer's feedback about the localization of GIVps4/46 in figures 1 and 2, we have included co-localization experiments using established organelle markers. For identifying posterior (PF), anterior (AF), and caudal flagella (CF), we utilized co-staining with alpha-tubulin, a well-known cytoskeletal marker that effectively labels these cytoskeletal structures (S1 Fig). This approach offers direct visual evidence of GIVps4/46 localization in relation to these cellular structures in fluorescence images. For peripheral vesicles (PVs), we used a previously reported protein which is known to reside in PVs, to confirm GIVps4/46 co-localization with PVs through dual immunofluorescence staining (S1 Fig) [1]. These modifications ensure that the localization of GIVps4/46 is not only based on their morphology through DIC but also on co-localization. Overall, the new images provide improved clarity and resolution, and the use of organelle-specific markers allows us to confidently assert the precise subcellular association of GIVps4/46 with these structures.

2. In figure 3 and related description (line 411-415), GlIst1 orthologs were found in some isolates, but not found in the other sequenced Giardia genomes. Also, later experiments were performed using WB strain. How do you think about the impact of the presence/absence of GlIst1 on Giardia lifecycles? Is it still essential for all Giardia, or important only for some Giardia strains?
Authors response:
It has been previously reported that Ist1 regulates the recruitment of Vps4 via Vps46 in yeast. Although ist1 deletion mutants do not have any observable defects on the ESCRT pathway, its loss in combination with other ESCRT components causes sorting defects mediated by ESCRT machinery, indicating that Ist1 is not essential but rather acts as a regulatory factor in ESCRT-mediated MVB biogenesis. Accordingly, Giardia strains lacking Ist1 may still carry out ESCRT functions, while strains that possess Ist1 likely use it to fine‑tune Vps4 recruitment through Vps46. This dispensable role of Ist1 may explain why Ist1 is not encoded by all Giardia strains.  This perspective has been included in the Discussion section of the revised manuscript (Line 661-668) 

3. In figure 3B, did authors confirm glist1 gene expression only in “WB isolate”? Please specify the strain used in the RT-PCR experiment.
Authors response:
We confirm that figure 3B exclusively represents the expression of the glist1 in the WB isolate. The RT-PCR analysis was conducted using RNA extracted from G. lamblia (ATCC 50803/WB clone C6). Line 417-419 in revised manuscript.

4. In figure 6 and related descriptions, they tried to address the effects of myristoylation on the cellular distribution of Ist1. GlIst1 was localized to both the endosome and the vacuole membrane, whereas ScIst1 was located within the endosome. Can you say distribution of GlIst1 was affected by myristoylation? The reviewer could not realize why myristoylation has the impact, not the other differences between Gl and Sc from the presented data.
Authors response:
To address the reviewer's comment regarding the potential role of myristoylation in the distribution of GlIst1, we generated point mutations at residue K43 (K→R, maintaining charge but eliminating the possibility of myristoylation, and K→A, altering charge and preventing myristoylation) and examined their localization in yeast. In both cases, the cellular distribution of GlIst1 (at the vacuolar membrane and endosomes) remained unchanged, although we observed reduced signal intensity in case of both mutants compared to wild type (please see the figure below).
These results suggest that the presence of a K at this position alone may not determine the distinct localization of GlIst1. However, since our experiments were performed in a heterologous yeast system, we are unable to draw any conclusions from these partially negative results.  Hence, we chose not to include them in the submitted version of the manuscript.  Longer-term studies, using localization of mutant GlIst1, may provide answers.  


Localization of GFP-GlIst1 wild-type (left panel); GFP-GlIst1K43R mutant (middle panel) and GFP-GlIst1K43A mutant (right panel). Scale bar represents 8 ìm. The images are obtained in same gain, numerical aperture, exposure time and magnification.

5. “Author summary” does not describe the importance of the study findings on Giardia lifecycle (stage-conversion), but just adds general knowledge about Giardiasis. Authors should explain how this work has an impact on the Giardia lifecycles.
Authors response:
We have revised the Author Summary to explicitly emphasize how our findings contribute to the understanding of the Giardia lifecycle, particularly the trophozoite-to-cyst stage-conversion. 

1. 	Datta SP, Jana K, Mondal A, Ganguly S, Sarkar S. Multiple paralogues of á-SNAP in Giardia lamblia exhibit independent subcellular localization and redistribution during encystation and stress. Parasites & Vectors. 2018 Oct 4;11(1):539.
