# Supplementary material for: Divergent functions of late ESCRT components in Giardia lamblia: Insights from subcellular distributions and protein interactions
Source: PLoS Negl Trop Dis. 2025 Nov 12;19(11):e0013700. doi: 10.1371/journal.pntd.0013700 (PMC12611102; doi:10.1371/journal.pntd.0013700)
Supplement: Attachment — Submitted filename: Response to Reviewer Patra et al.rtf [file pntd.0013700.s015.rtf]

1. Line 452-453: 'However, an MIT-like domain must be present in GlVps4b as
453 it can functionally substitute for ScVps4'. How can this be true if in yeast two hybrid screening they could not substitute each other. But if it they functionally complement as per Ref 25 cited then why do they not complement each other in the yeast two hybrid assay.
Our response: Based on two independent assays (growth in presence of LiCl and trafficking of GFP-CPS to the vacuole lumen), we had previously reported that GlVps4b can functionally substitute for ScVps4 in vivo. However, Y2H experiments of our present study documents that, unlike the MIT domain of ScVps4, the non-canonical MIT domain of GlVps4b does not directly interact with ScIst1. This apparent incongruity may be explained taking into account the difference between what these two assays evaluate: while Y2H detects direct binary interaction between a pair of proteins, the two complementation assays gauge the ability of GlVps4b to function as a component of the ESCRT pathway in yeast.  Thus, the latter assays are a readout of the full spectrum of Vps4 activities—including interaction with multiple ESCRT-III subunits and regulators, ability of Vps4 to assemble into a hexamer, and also carry out ATP hydrolysis, all of which encompass a wider assessment of Vps4 activity, beyond any single binary MIT-MIM interaction.
Previous studies in yeast have shown that Vps4 recruitment to ESCRT-III involves multiple overlapping MIM-MIT interactions with subunits such as Vps2, Vps20, Did2, and Vta1, which act cooperatively and redundantly [1]. Such parallel pathways for Vps4 recruitment is established by the fact that although ist1 deletion mutants do not have any observable defects on the ESCRT pathway, loss of Ist1 function in combination with other ESCRT components causes defects in ESCRT-mediated sorting events [2]. Therefore, while GlVps4b's MIT domain may not bind directly to ScIst1, alternate interactions with other yeast ESCRT-III components are likely to result in its recruitment to sites of ESCRT activity. Hence, loss of one specific MIT-MIM interaction may not significantly abrogate overall ESCRT function. Together, these observations underscore the complexity and redundancy of Vps4-ESCRT-III interactions and suggest that the functional complementation observed in yeast is possible even without the MIT-MIM interaction between GlVps4b and ScIst1. 

2. It is unclear how the construct GlVps4b1-191 or GIVps46b were expressed inside E. coli for co-purification studies. Same for GlVps4b1-109, GlVps4b30-109 for b-galactosidase assays.
Our response: We have provided these details in the revised manuscript (Line no.150-161).

3. It is strange to find that GlVps4b has an non-canonical MIT domain but its interacting partner GIIst1 has a canonical MIM domain predicted through AlphaFold and the same interacting with non-canonical MIT of GIVps4b. Besides if GIIst1 has canonical MIM domain identified by AlphaFold then why doesn't it interact with canonical MIT domain in ScVps4.
Our response: In the manuscript, we have not categorized the MIM in GlIst1 as a canonical MIM. As mentioned in the text (Line no. 496-498), “Similar to the canonical MIMs in the yeast and human orthologues, this helix is located near the C-terminal end of GlIst1, and it contains both hydrophobic (I305, L309, L312, and Y315) and positively charged (R310) residues (Fig 4F)”.  Hence, while the position of this helix is at the C-terminal, as in other Ist1 orthologs, its sequence does not conform to the sequence of canonical MIMs. 
In this context, it is also worth noting that literature documents that although all twelve human ESCRT-III proteins have MIMs at their C-terminus, their binding affinity for various MIT domains differ significantly. These interactions range from very tight binding to moderate, weak, or even no detectable binding. This diversity in binding affinity establishes that the interaction between MIT and MIM is not uniform, but is a result of a complex binding code determined by the number and type of MIMs, their structural characteristics and the variety of binding modes they can adopt [3,4]

4. What is meant by an N-terminal Ist1 domain in a GIst1 protein. Is this domain at the N-terminus also non-canonical to similar proteins of other species.
Our response: Our manuscript mentions that domain prediction tools detect an Ist1 domain in GlIst1, even though this protein has very low sequence identity with the yeast and human orthologs (Line no. 547-549).  As stated in the manuscript, this predicted domain in GlIst1 is smaller than the Ist1 domains of the yeast and human proteins. Pairwise alignment also reveals that the identified Ist1 domain of GlIst1 shares only 11.6% identity and 22.2% similarity with the domain in ScIst1 and 15.0 % identity and 25.2% similarity with the domain in the human orthologue.  Besides these low identity values, the reduced size of the Ist1 domain in GlIst1, further indicate its non-canonical nature. Future structure-function characterizations will identify key residues within this domain. The relevant section has been revised accordingly in the updated manuscript (Line no. 551-555).

5. Since GlIst1 interacts with GIVps4b via the C-terminus and with GIVps46b with the N-terminus it would be interesting to find if they can be co-purified together as a complex which then would indicate that GIst1 acts as the seeding protein glueing the GIVps proteins together at the membrane.
Our response: We appreciate the Reviewer's insightful suggestion to examine the potential co-purification of GlIst1, GlVps4b, and GlVps46b as a ternary complex. Presence of such a trimer will be established if immunoprecipitation with any one of the three proteins brings down the other two.  While we have antibodies specific to GlVps4b and GlVps46b, we do not have an anti-GlIst1 antibody at present. We are in the process of generating this tool, which will also allow us to carry out co-localization experiments.

6. Since GIst1 and ScIst1 do not complement each other the homologous localization of GIst1 in Giardia is more important over its heterologous expression and co-localization with ScIst1 in yeast.
Our response: We acknowledge the Reviewer's suggestion. As mentioned previously, we are raising antibody specific to GlIst1, which will allow us to localize this protein in Giardia.

7. Were the proteomic profiling of GlIst1 carried out using mass spectrometric analysis or was it predicted through bioinformatic tools. It would be good to validate it through mass spectrometric analysis.
Our response: We would like to clarify that, as stated in the Methods section, the proteomic profiling of GlIst1 was not based on any bioinformatic prediction, but was carried out using LC-ESI-MS/MS analysis from both Giardia trophozoites and 16 h encysting cells. The data is given in Supplementary Figure S7. We have now revised the text in the Results section to explicitly state that these PTMs were validated through mass spectrometry, in order to avoid any possible ambiguity (Line no. 605-606).

8. Could the myristoylation of K43 of GlIst1 be important for its interaction with GIVps46b.
Our response: It has been previously documented that myristoylation contributes to the protein-protein interaction such as neuronal protein NAP-22 of rat binds calmodulin in a myristoylation-dependent manner through the hydrophobic myristoyl group interacting specifically with calmodulin's hydrophobic pocket. Loss or inhibition of myristoylation abolishes this interaction [5]. Another report also suggests interaction between two mitochondrial proteins FSP1 and TOMM40 is dependent on myristoylation of FSP1 [6].
Thus, it is possible that myristoylation at K43 residue of GlIst1 is important for its interaction with GlVps46b and mutation of the myristoylation site will weaken or abolish the interaction. Our future work will focus on directly testing this hypothesis by mutating K43 to non-myristoylable residue(s) and assessing the consequent effects on GIIst1's binding to GIVps46b through co-immunoprecipitation in Giardia. Additionally, we aim to examine changes in subcellular localization and membrane association due to loss of myristoylation, which may reveal altered interaction dynamics.
1. 	Azmi IF, Davies BA, Xiao J, Babst M, Xu Z, Katzmann DJ. ESCRT-III Family Members Stimulate Vps4 ATPase Activity Directly or via Vta1. Developmental Cell. 2008 Jan;14(1):50–61. 
2. 	Dimaano C, Jones CB, Hanono A, Curtiss M, Babst M. Ist1 Regulates Vps4 Localization and Assembly. Lemmon S, editor. MBoC. 2008 Feb;19(2):465–74. 
3. 	Wenzel DM, Mackay DR, Skalicky JJ, Paine EL, Miller MS, Ullman KS, et al. Comprehensive analysis of the human ESCRT-III-MIT domain interactome reveals new cofactors for cytokinetic abscission. eLife. 2022 Sept 15;11:e77779. 
4. 	Lee S, Chang J, Renvoisé B, Tipirneni A, Yang S, Blackstone C. MITD1 is recruited to midbodies by ESCRT-III and participates in cytokinesis. Lemmon S, editor. MBoC. 2012 Nov 15;23(22):4347–61. 
5. 	Taniguchi H. Protein myristoylation in protein–lipid and protein–protein interactions. Biophysical Chemistry. 1999 Dec;82(2–3):129–37. 
6. 	Fedoryshchak RO, Gorelik A, Shen M, Shchepinova MM, Pérez-Dorado I, Tate EW. Discovery of lipid-mediated protein–protein interactions in living cells using metabolic labeling with photoactivatable clickable probes. Chem Sci. 2023;14(9):2419–30.
